# Online self-regulated English learning and English learning anxiety in digital contexts: the mediating role of intrinsic motivation

**DOI:** 10.3389/fpsyg.2026.1797013

**Published:** 2026-05-18

**Authors:** Xiaosa Wang, Lin Luo

**Affiliations:** 1 School of Education and Foreign Languages, Wuhan Donghu University, Wuhan, Hubei, China; 2 School of Physical Education, Guizhou Normal University, Guiyang, Guizhou, China; 3 Key Laboratory of Brain Function and Brain Science of Guizhou Province, Guiyang, Guizhou, China

**Keywords:** digital learning environments, English learning anxiety, intrinsic motivation, online self-regulated learning, self-determination theory

## Abstract

**Background:**

With the rapid digital transformation of higher education, online self-regulated English learning (OSRL) has become increasingly important; however, its emotional consequences, particularly English learning anxiety, remain insufficiently understood. In these digital learning environments, students are expected to regulate their learning processes while also coping with emotional challenges such as English learning anxiety. Nevertheless, little is known about how different dimensions of OSRL relate to anxiety and whether intrinsic motivation may explain these associations.

**Methods:**

A cross-sectional design was employed, and 1,361 valid responses were collected from university students in China using the Maike Online Questionnaire Platform. Three validated instruments were used: the Online Self-Regulated Learning Questionnaire (OSLQ) to assess six dimensions of learners' self-regulatory behavior, the English Class Performance Anxiety Questionnaire (ECPAQ) to measure anxiety in English learning contexts, and the Intrinsic Motivation subscale of the Language Learning Orientations Scale (LLOS-IM) to assess intrinsic motivation. Data were analyzed using Spearman correlation and bootstrap-based multiple mediation analysis (5,000 resamples) to examine the direct and indirect associations between online self-regulated English learning dimensions and English learning anxiety.

**Results:**

The findings showed that different dimensions of online self-regulated English learning were differentially associated with English learning anxiety. Help-seeking significantly predicted lower anxiety levels (β = −0.569, *p* = 0.006), suggesting that social support may serve as a protective factor in digital English learning contexts. Self-evaluation showed a suppression-like indirect pattern (i.e., indirect and direct effects operating in opposite directions) (indirect a × b = 0.116, accounting for 22.65% of the total effect), indicating that while self-monitoring enhances learning control, it may also amplify performance pressure and emotional strain. In contrast, goal setting exerted a fully mediated effect through intrinsic motivation (a × b = 0.234, accounting for 100% of its total effect), showing that motivational quality is a key determinant of whether self-regulatory behaviors reduce or exacerbate anxiety.

**Conclusion:**

This study suggests that online self-regulated learning does not uniformly alleviate English learning anxiety; rather, its emotional associations depend on the interaction between intrinsic motivation and social-cognitive factors. While excessive self-evaluation may trigger anxiety through heightened self-expectation, adaptive goal setting can enhance intrinsic motivation and indirectly mitigate anxiety. These findings highlight the importance of considering motivational quality and contextual factors when interpreting the emotional outcomes of self-regulated learning, and offer empirical guidance for designing low-anxiety, motivation-enhancing digital learning environments for English language instruction.

## 1 Introduction

With the deepening digital transformation of higher education in China, online and blended English teaching models have become an essential component of foreign language education. As the pedagogical paradigm shifts from teacher-centered to learner-centered approaches, students in online learning environments are increasingly required to develop strong self-regulated learning (SRL) abilities—that is, the capacity to actively plan, monitor, and reflect upon their own learning processes (Zimmerman, 1989, 2002). The Action Plan for the Digital Transformation of Higher Education (Ministry of Education of the People's Republic of China 2022) and the Education Informatization 2.0 Action Plan (Ministry of Education of the People's Republic of China, 2018) both emphasize the construction of a learner-centered digital learning ecosystem, encouraging learners to become active agents in their learning process. This shift indicates that online self-regulated learning has become a key psychological variable influencing not only learning outcomes but also emotional experiences such as English learning anxiety (Huang et al., 2020).

According to Social Cognitive Theory, self-regulated learners are capable of setting appropriate learning goals, applying effective strategies, and regulating both motivation and emotions, thereby optimizing their academic performance (Schunk and Zimmerman, 2011). Previous studies have shown that students with higher levels of self-regulation tend to employ metacognitive and effort regulation strategies more effectively, displaying higher engagement and better academic achievement in online contexts (Broadbent and Poon, 2015; Theobald, 2021). Empirical research conducted in China and other Asian regions has further demonstrated that online self-regulation is closely related to learning motivation and self-efficacy, and facilitates English learning outcomes through positive learning behaviors (Zheng et al., 2018; Apridayani et al., 2023). For example, (Rui and Liu 2023) found that perceived teacher support positively predicted online English learners' self-regulation through self-efficacy, suggesting that motivational and contextual factors are closely intertwined with self-regulated learning in digital English education. These findings indicate that SRL operates within a broader motivational and contextual system, rather than as an isolated cognitive process. However, compared with its effect on academic achievement, the mechanisms through which online self-regulated learning influences English learning anxiety remain insufficiently explored (Soodmand Afshar, 2020).

English learning anxiety (ELA) is recognized as a critical affective variable that significantly influences second language acquisition. It can impair attentional control, lower self-confidence, and inhibit linguistic output, thereby hindering learning performance (Horwitz, 1986; Zeidner, 2007; Raver et al., 2007). Traditional studies have largely focused on the direct negative impact of anxiety, with relatively few examining how self-regulated learning and motivational factors interact to shape emotional outcomes. Recent findings suggest that although self-regulated learning may alleviate anxiety, its impact is contingent on the nature or quality of learners' motivation (Özer and Akçayoglu, 2021; Bai and Wang, 2023). Specifically, high motivation is not inherently beneficial; in performance-driven or externally evaluative contexts, it may transform into a source of pressure, thereby triggering anxiety rather than reducing it. Therefore, a key unresolved issue is whether and how motivational processes mediate the relationship between self-regulated learning and anxiety in digital learning contexts.

Drawing upon Self-Determination Theory (SDT), intrinsic motivation is viewed as a relatively autonomous form of motivation that is typically associated with enjoyment, engagement, and adaptive learning outcomes. In the present study, SDT serves as the primary theoretical framework to explain the mediating role of intrinsic motivation, while Social Cognitive Theory provides the foundation for understanding self-regulated learning processes. When intrinsic motivation stems from autonomy and competence, it fosters positive emotions, deeper engagement, and long-term persistence. However, in highly evaluative learning contexts, even relatively autonomous engagement may coexist with strong self-expectation, social comparison, and concern about performance, which may in turn be associated with anxiety. Achievement Goal Theory and Control-Value Theory are incorporated as complementary perspectives to explain why motivationally engaged learners may still experience anxiety under conditions of heightened evaluative pressure, rather than as parallel core frameworks. From this perspective, self-regulated learning may be associated with lower anxiety through enhanced control and engagement, but some self-regulatory processes may also coincide with greater pressure, yielding an indirect/direct sign inconsistency that may reflect a model-dependent suppressor-like pattern rather than a uniformly protective association. Importantly, this pattern should not be interpreted as evidence against Self-Determination Theory itself, but rather as a context-specific statistical association observed under the simultaneous inclusion of correlated predictors.

Moreover, different dimensions of self-regulated learning may influence anxiety through distinct psychological pathways. The self-evaluation dimension reflects learners' monitoring and reflection on their progress. While this process can enhance a sense of mastery and control, it may simultaneously increase performance pressure, thereby producing an indirect/direct sign inconsistency (i.e., the indirect and direct effects operate in opposite directions), which may reflect a model-dependent suppressor-like pattern rather than a definitive suppression effect between motivation and anxiety. Conversely, the goal-setting dimension, which is closely linked to the activation of intrinsic motivation, may exert a fully mediating influence on emotional outcomes by enhancing motivational quality. Empirical findings provide preliminary support for these assumptions. Apridayani et al. (2023) reported that self-regulated learning strategies significantly predicted academic achievement in online English courses, yet the association between anxiety and achievement was non-significant—suggesting that motivation may mask the influence of anxiety. Similarly, Zheng et al. (2018) found that online self-regulation indirectly affected learning outcomes through motivational mechanisms, while Soodmand Afshar (2020) identified a complex interplay between self-regulation and anxiety in online oral performance.

In summary, the present study constructs and examines a mediation model of online self-regulated English learning dimensions (e.g., self-evaluation, goal setting) → intrinsic motivation for English learning → English learning anxiety within the context of China's digital higher education reform. Specifically, this study investigates how different self-regulation components are associated with anxiety through motivational quality. The following hypotheses are proposed: (1) online self-regulated learning is negatively associated with English learning anxiety; and (2) it is indirectly associated with English learning anxiety via intrinsic motivation, with potentially different indirect patterns across dimensions. More specifically, self-evaluation is expected to show an indirect/direct sign inconsistency that may reflect a model-dependent suppressor-like pattern, whereas goal setting is expected to show a fully mediated association through intrinsic motivation. By focusing on the heterogeneity of SRL dimensions and their distinct motivational pathways, this study aims to extend existing research by demonstrating that self-regulated learning does not exert uniform emotional effects. This study aims to elucidate the complex interplay among self-regulation, motivation, and emotion in the era of digital learning, and to provide empirical evidence for designing low-anxiety, high-motivation online environments that enhance learners' wellbeing and language achievement.

## 2 Methods

### 2.1 Data source

This cross-sectional study was conducted between March and April 2023. Data were collected via the Maike online survey platform, and the questionnaire link (QR code) was disseminated through the social media platform WeChat. A convenience sampling strategy was adopted, and university students participated voluntarily by scanning the code and completing the questionnaire after providing informed consent.

Participants were eligible for inclusion if they met the following criteria: (i) aged between 18 and 29 years; (ii) capable of independently using digital media tools; and (iii) enrolled in universities offering English courses (either compulsory or elective). Responses were excluded if: (i) the participant's age fell outside the specified range; or (ii) implausible responses were provided (e.g., reporting the study of a non-English foreign language in an English-specific context).

The study received ethical approval from the Ethics Committee of the School of Physical Education, Guizhou Normal University (Approval No. GZNUPEI20220524), and all procedures adhered to the latest revision of the Declaration of Helsinki. All participants were informed of the voluntary nature of the study and their right to withdraw at any time without penalty.

Of the 1,421 students who initially completed the survey, 1,361 valid responses were retained based on the inclusion and exclusion criteria. These participants, drawn from various provinces in China, had a mean age of 18.94 ± 1.07 years, and females comprised 73.84% of the final sample. The sample was characterized by a relatively high proportion of female students and freshmen, reflecting an uneven demographic distribution that may limit representativeness and should be considered when interpreting the findings. This demographic imbalance may also influence the generalizability of the results, particularly because English learning anxiety and self-regulated learning patterns may vary across gender and academic year. Therefore, the findings should be interpreted cautiously when extending them to more balanced or diverse university populations. Detailed demographic characteristics are presented in Table 1.

**Table 1:** Demographic characteristics of participants.

Variables	Total (*n* = 1,361)	Male (*n* = 356)	Female (*n* = 1,005)	*p*-value
Age (years, Mean ± SD)	18.94 ± 1.07	19.19 ± 1.22	18.86 ± 1.01	< 0.001
Household registration, *n* (%)				0.005
Rural	695 (51.07)	182 (51.12)	513 (51.04)	
Urban	402 (29.54)	87 (24.44)	315 (31.34)	
City	264 (19.40)	87 (24.44)	177 (17.61)	
Self-assessment of English proficiency, *n* (%)				< 0.001
Very poor	489 (35.93)	167 (46.91)	322 (32.04)	
Poor	474 (34.83)	105 (29.49)	369 (36.72)	
Normal	370 (27.19)	72 (20.22)	298 (29.65)	
Good	17 (1.25)	6 (1.69)	11 (1.09)	
Excellent	11 (0.81)	6 (1.69)	5 (0.50)	
Grade level, *n* (%)				< 0.001
Freshman	1,244 (91.40)	311 (87.36)	933 (92.84)	
Sophomore	94 (6.91)	41 (11.52)	53 (5.27)	
Junior	21 (1.54)	3 (0.84)	18 (1.79)	
Senior	2 (0.15)	1 (0.28)	1 (0.10)	

SD, Standard deviation. p-values were obtained from independent samples t-tests or chi-square tests as appropriate.

### 2.2 Measures

#### 2.2.1 Demographic information

Participants provided demographic data, including gender (male, female), age, household registration type (rural, urban, city), academic year (freshman, sophomore, junior, senior), and self-assessed English proficiency (very poor, poor, normal, good, excellent).

#### 2.2.2 English class performance anxiety

English learning anxiety was assessed using the English Class Performance Anxiety Questionnaire developed by Aslan and Thompson (2021). The scale was translated and adapted following a standard translation–back translation protocol Brislin, (1970). Two independent translators initially translated the items into Chinese, and the translation was reviewed by five bilingual experts. Two additional translators conducted a back translation into English, which was further revised through expert consultation in educational psychology. The Chinese version of the scale had previously demonstrated good reliability and validity in pilot studies.

The scale comprises 16 items measuring performance-related anxiety in English classrooms (e.g., “I always feel that the other students speak English better than I do.”). Responses are rated on a 5-point Likert scale (1 = “Strongly disagree” to 5 = “Strongly agree”), with higher scores indicating greater anxiety. In the present study, the internal consistency of the scale was excellent (Cronbach's α = 0.945). To further establish construct validity in the current sample, confirmatory factor analysis (CFA) was conducted, and both convergent validity and discriminant validity were evaluated using standardized factor loadings, composite reliability (CR), and average variance extracted (AVE), following recommended psychometric guidelines.

#### 2.2.3 Online self-regulated English learning

Online self-regulated English learning was measured using the Online Self-Regulated Learning Questionnaire (OSLQ) developed by Barnard et al. (2009). The original scale consists of 24 items across six dimensions: Goal Setting (5 items), Environmental Structuring (4 items), Task Strategies (4 items), Time Management (3 items), Help Seeking (4 items), and Self-Evaluation (4 items). The translation and adaptation process followed the same rigorous translation–back translation procedures described above.

In this study, the revised Chinese version retained 23 items after removing one item from the Self-Evaluation dimension for cultural adaptation. This modification was made to improve contextual appropriateness without altering the conceptual structure of the scale. All other dimensions remained unchanged. To ensure contextual relevance, all items were modified to explicitly reflect the context of English learning (e.g., specifying “in your English online study” within each item). Responses were recorded on a 5-point Likert scale (1 = “Strongly disagree” to 5 = “Strongly agree”), with higher scores reflecting greater self-regulation in online English learning. The scale demonstrated excellent reliability in this sample (Cronbach's α = 0.967). The factorial validity of the revised Chinese version was further supported by CFA results. In addition, convergent and discriminant validity were assessed to confirm that the six dimensions represented empirically distinguishable, though related, components of online self-regulated English learning.

Additionally, to explore the distinct contributions of each self-regulation dimension to English learning anxiety, the six subscales were analyzed independently in subsequent mediation models.

#### 2.2.4 Intrinsic motivation for English learning

Intrinsic motivation for English learning was measured using the Intrinsic Motivation Subscale of the Language Learning Orientations Scale (LLOS-IM) developed by Noels et al. (2000). This subscale includes 7 items (e.g., “I enjoy learning English.”) that assess learners' interest and enjoyment in learning English. Each item is rated on a 5-point Likert scale (1 = “Strongly disagree” to 5 = “Strongly agree”), with higher scores indicating stronger intrinsic motivation. The Chinese version of the scale demonstrated good internal consistency (Cronbach's α = 0.895). CFA was conducted to further evaluate the factorial structure of this subscale, and its convergent validity was assessed using CR and AVE in accordance with established criteria.

### 2.3 Statistical analysis

All statistical analyses were conducted using SPSS version 26.0 (IBM Corp., Armonk, NY, USA). To assess common method bias, Harman's single-factor test was first applied. The results showed that the first principal component accounted for less than 40% of the total variance, suggesting that common method variance was unlikely to be a dominant concern. However, as Harman's single-factor test alone is insufficient to definitively rule out common method bias, the findings should be interpreted with caution. In addition, several procedural remedies were adopted during data collection, including anonymous participation, voluntary response, and standardized instructions, to reduce potential method-related bias. Nevertheless, given the self-report and cross-sectional design, the possibility of residual common method bias and self-selection bias cannot be entirely excluded.

Descriptive statistics were computed for all main study variables, including sample distributions, means, and standard deviations. The Shapiro–Wilk test was used to assess the normality of continuous variables (age, English class anxiety, online self-regulated English learning, and intrinsic motivation). Due to violations of normality assumptions for several variables, Spearman's rank-order correlation coefficients were used for bivariate analyses.

According to Cohen (2013), the strength of correlation is interpreted as follows: *r* ≤ 0.19 = negligible; 0.20–0.39 = low; 0.40–0.59 = moderate; 0.60–0.79 = high; ≥ 0.80 = very high. The internal consistency of each scale was evaluated using Cronbach's alpha, with α > 0.70 considered acceptable (Sawyer et al., 2014). To provide more robust psychometric evidence, CFA was conducted using AMOS version 26.0. Model fit was evaluated using multiple indices, including the comparative fit index (CFI), Tucker–Lewis index (TLI), root mean square error of approximation (RMSEA), and standardized root mean square residual (SRMR). Values of CFI and TLI above 0.90 and RMSEA and SRMR below 0.08 were considered indicative of acceptable fit. Convergent validity was considered adequate when standardized factor loadings were significant and preferably above 0.50, CR exceeded 0.70, and AVE exceeded 0.50. Discriminant validity was examined by comparing the square root of the AVE for each construct with its inter-construct correlations.

Mediation analysis was conducted using a multiple regression-based approach (PROCESS framework). In Model 1, the independent variable (online self-regulated English learning and its dimensions) was used to predict the dependent variable (English class anxiety). In Model 2, the independent variable predicted the mediator (intrinsic motivation). In Model 3, both the independent variable and the mediator were entered simultaneously to predict the dependent variable. Total, direct, and indirect effects were estimated. Given the relatively high correlations among several self-regulated learning dimensions, multicollinearity diagnostics were conducted prior to model estimation. Variance inflation factor (VIF) and tolerance statistics were examined, with VIF values below 5.0 and tolerance values above 0.20 indicating that multicollinearity was not severe enough to bias the regression estimates.

Because formal suppressor diagnostics were not conducted, the mediation analysis focused on identifying indirect/direct sign inconsistencies and model-dependent association patterns rather than establishing a definitive statistical suppression effect. Therefore, any suppressor-like interpretation in the present study should be understood cautiously as an exploratory explanatory framework rather than a formally verified statistical conclusion.

Following Hayes (2022), bootstrap resampling (5,000 iterations) was used to generate 95% confidence intervals (CIs) for all indirect effects. An effect was considered significant if the CI did not include zero (Rijnhart et al., 2021; Hayes, 2022). The significance level was set at *p* < 0.05 (two-tailed). To facilitate interpretation of the practical magnitude of the findings, the explained variance (R^2^) for each regression model was reported, and changes in R^2^ after including the mediator were additionally examined as indicators of effect size. Given the relatively large sample size, statistical significance was interpreted alongside effect size indicators to avoid overestimating the practical importance of modest associations.

## 3 Results

### 3.1 Descriptive statistics of key variables

Table 2 summarizes the means, standard deviations, and Shapiro–Wilk test results for the primary variables in this study. The test results showed that none of the variables conformed to a normal distribution, with all *p-values* less than 0.001. These findings indicate significant violations of the normality assumption and thus justify the use of non-parametric statistical methods in subsequent analyses. Accordingly, non-parametric correlation coefficients were adopted to ensure the robustness of the results under non-normal conditions.

**Table 2:** Descriptive statistics and normality test results for key variables (*N* = 1,361).

Variable	Mean	SD	Shapiro–Wilk W	
			W-value	*p*-value
Self-evaluation	9.256	2.257	0.903	< 0.001
Help seeking	12.444	2.772	0.919	< 0.001
Time management	9.424	2.233	0.898	< 0.001
Task strategies	12.31	2.816	0.913	< 0.001
Environmental structuring	13.163	2.875	0.909	< 0.001
Goal setting	15.655	3.741	0.93	< 0.001
Intrinsic motivation for English learning	2.845	0.761	0.957	< 0.001
English class performance anxiety	50.665	10.755	0.954	< 0.001

### 3.2 Correlation analysis among variables

Table 3 presents the Spearman correlation coefficients among the main study variables. The results indicate that the majority of variable pairs were significantly correlated at the *p* < 0.01 level, with several correlations also reaching significance at the *p* < 0.05 level. The six dimensions of online self-regulated English learning were moderately to highly intercorrelated (*r* = 0.542 to 0.755, all *p* < 0.01), suggesting substantial conceptual overlap among these components. This pattern further justifies the need for multicollinearity diagnostics in subsequent regression analyses.

**Table 3:** Spearman correlation coefficients among key variables (*N* = 1,361).

Variable	1	2	3	4	5	6	7
1 Self-evaluation	1						
2 Help seeking	0.755^**^	1					
3 Time management	0.686^**^	0.682^**^	1				
4 Task strategies	0.655^**^	0.654^**^	0.737^**^	1			
5 Environmental structuring	0.566^**^	0.570^**^	0.654^**^	0.629^**^	1		
6 Goal setting	0.573^**^	0.542^**^	0.663^**^	0.632^**^	0.680^**^	1	
7 Intrinsic motivation for English learning	0.365^**^	0.334^**^	0.395^**^	0.387^**^	0.395^**^	0.480^**^	1
8 English class performance anxiety	0.085^**^	0.102^**^	0.060^*^	0.078^**^	0.085^**^	0.035	−0.171^**^

^*^p < 0.05; ^**^p < 0.01.

Intrinsic motivation for English learning was positively correlated with all six self-regulated learning dimensions (*r* = 0.334 to 0.480, all *p* < 0.01), whereas it was negatively correlated with English class performance anxiety (*r* = −0.171, *p* < 0.01). This negative bivariate association contrasts with the positive regression coefficient observed in later multivariate models, suggesting an indirect/direct sign inconsistency that may reflect a model-dependent suppressor-like pattern when multiple correlated predictors are considered simultaneously, rather than definitive evidence of a formal statistical suppression effect.

In contrast, the bivariate correlations between the self-regulated learning dimensions and English class performance anxiety were small in magnitude, and goal setting was not significantly correlated with anxiety (*r* = 0.035, *p* > 0.05). Given the relatively high intercorrelations among several predictors, multicollinearity diagnostics were subsequently examined in the regression-based mediation analyses.

### 3.3 Mediating effects: regression analysis results

Table 4 presents the results of a bootstrapped (5,000 samples) multiple regression-based mediation analysis. In Model 1, the six dimensions of English online self-regulated learning were included as independent variables, with English learning anxiety as the dependent variable. Model 2 treated intrinsic motivation for English learning as the dependent variable. Model 3 simultaneously included intrinsic motivation and the six self-regulated learning dimensions to predict English learning anxiety. Before estimating the mediation models, multicollinearity diagnostics were conducted for the predictors entered simultaneously into the regression equations, and all VIF values were within acceptable ranges, indicating that multicollinearity did not substantially bias parameter estimation.

**Table 4:** Bootstrap (5,000 samples) regression results for mediation analysis.

Predictor	*B*	SE	*t*	*p*	β
Model 1: English learning anxiety (Y)					
Self-evaluation	−0.397	0.232	−1.716	0.086	−0.083
Help seeking	−0.569^**^	0.206	−2.764	0.006	−0.147
Time management	0.504	0.262	1.924	0.055	0.105
Task strategies	−0.046	0.203	−0.225	0.822	−0.012
Environmental structuring	−0.200	0.175	−1.142	0.254	−0.053
Goal setting	0.060	0.132	0.455	0.649	0.021
*R^2^* = 0.032 Adjusted *R^2^* = 0.028 F_(6, 1354)_ = 7.464, *p* < 0.001					
Model 2: Intrinsic motivation for English learning (M)					
Self-evaluation	0.033^*^	0.014	2.286	0.022	0.098
Help seeking	−0.007	0.013	−0.527	0.598	−0.025
Time management	0.009	0.016	0.568	0.570	0.027
Task strategies	0.020	0.013	1.618	0.106	0.076
Environmental structuring	0.014	0.011	1.272	0.204	0.052
Goal setting	0.066^**^	0.008	8.077	< 0.001	0.325
*R^2^* = 0.253 Adjusted *R^2^* = 0.250 F_(6, 1354)_ = 76.446, *p* < 0.001					
Model 3: English learning anxiety (Y, with intrinsic motivation included)					
Self-evaluation	−0.514^*^	0.226	−2.268	0.023	−0.108
Help seeking	−0.545^**^	0.201	−2.714	0.007	−0.140
Time management	0.471	0.256	1.844	0.065	0.098
Task strategies	−0.118	0.198	−0.594	0.552	−0.031
Environmental structuring	−0.249	0.171	−1.455	0.146	−0.066
Goal setting	−0.174	0.132	−1.321	0.187	−0.061
Intrinsic motivation for English learning	3.535	0.427	8.285	< 0.001	0.250
*R^2^* = 0.079 Adjusted *R^2^* = 0.074 F_(7, 1353)_ = 16.524, *p* < 0.001					

^*^p < 0.05; ^**^p < 0.01. B, Unstandardized regression coefficient; SE, Standard error; β, Standardized regression coefficient.

In Model 1, Help Seeking showed a significant negative association with English learning anxiety (*B* = −0.569, *p* = 0.006), while Self-Evaluation exhibited a marginally significant negative effect (*B* = −0.397, *p* = 0.086). The remaining dimensions did not yield significant effects. This model accounted for a small proportion of variance in English learning anxiety (*R*^2^ = 0.032). This relatively low explanatory power suggests that self-regulated learning dimensions alone may not sufficiently account for variability in anxiety, and that additional motivational or contextual factors are likely involved.

In Model 2, both Self-Evaluation (*B* = 0.033, *p* = 0.022) and Goal Setting (*B* = 0.066, *p* < 0.001) significantly and positively predicted intrinsic motivation for English learning. The model demonstrated substantial explanatory power (*R*^2^ = 0.253).

In Model 3, intrinsic motivation emerged as a significant positive predictor of English learning anxiety (*B* = 3.535, *p* < 0.001). Importantly, both Help Seeking (*B* = −0.545, *p* = 0.007) and Self-Evaluation (*B* = −0.514, *p* = 0.023) remained significantly negatively associated with anxiety. This model explained a modest portion of variance (*R*^2^ = 0.079). Compared with Model 1, the inclusion of intrinsic motivation increased the explained variance in English learning anxiety from 3.2% to 7.9%, indicating that motivational processes contributed additional explanatory value, although the overall effect size remains modest and should be interpreted cautiously. Given the relatively large sample size, statistical significance should not be interpreted as indicating strong practical effects. Rather, the findings suggest theoretically meaningful but practically modest associations, and other contextual factors may play equally important roles in explaining English learning anxiety.

In summary (see Table 5), intrinsic motivation for English learning was statistically associated with the relationship between certain facets of online self-regulated learning and English learning anxiety—functioning as a model-dependent indirect/direct sign inconsistency for Self-Evaluation and as a full mediator for Goal Setting. These findings indicate that the role of intrinsic motivation is not uniformly facilitative; rather, its function depends on the interplay between different self-regulatory processes and model specifications. Notably, the positive regression coefficient of intrinsic motivation observed in Model 3 should be interpreted in the context of its negative bivariate correlation with anxiety, suggesting a model-dependent statistical association under the simultaneous inclusion of correlated predictors rather than evidence that intrinsic motivation itself increases anxiety or contradicts Self-Determination Theory. These findings should be interpreted as associative patterns rather than definitive causal relationships.

**Table 5:** Summary of mediation pathways and effect sizes.

Mediation pathway	Mediation type	Total effect (c)	Indirect effect (a × b)	Direct effect (c^′^)	Proportion mediated
Self-Evaluation → Intrinsic Motivation for English Learning → English Class Performance Anxiety	Suppression-like indirect pattern	−0.397	0.116	−0.514	22.65%
Help Seeking → Intrinsic Motivation for English Learning → English Class Performance Anxiety	Non-significant	−0.569	−0.024	−0.545	0%
Time Management → Intrinsic Motivation for English Learning → English Class Performance Anxiety	Non-significant	0.504	0.033	0.471	0%
Task Strategies → Intrinsic Motivation for English Learning → English Class Performance Anxiety	Non-significant	−0.046	0.072	−0.118	0%
Environmental Structuring → Intrinsic Motivation for English Learning → English Class Performance Anxiety	Non-significant	−0.200	0.049	−0.249	0%
Goal Setting → Intrinsic Motivation for English Learning → English Class Performance Anxiety	Full mediation	0.060	0.234	−0.174	100%

Model-dependent indirect/direct sign inconsistency refers to cases where the indirect and direct effects operate in opposite directions and may reflect a suppressor-like statistical pattern, although no formal suppressor diagnostics were conducted. Full mediation refers to a non-significant direct effect with a significant indirect effect.

## 4 Discussion

This study, set against the backdrop of digitalized English instruction in Chinese universities, examined the associations through which six dimensions of online self-regulated learning influence English Class Performance Anxiety via Intrinsic Motivation for English Learning. The findings revealed that the dimension of Self-Evaluation exhibited a model-dependent indirect/direct sign inconsistency, Goal Setting demonstrated full mediation, and Help Seeking directly and negatively predicted anxiety. These results indicate that self-regulated learning is not a uniformly positive construct; its emotional outcomes depend on the quality of motivation and the availability of social support. This supports the proposition in Self-Determination Theory (SDT) by Ryan and Deci (as cited in John et al., 2010) that the quality rather than the intensity of motivation plays a more crucial role in shaping learners' emotional experiences. Importantly, the present findings extend this perspective by demonstrating that the effects of self-regulated learning on anxiety are dimension-specific and operate through differentiated motivational pathways rather than a uniform mechanism.

The model-dependent indirect/direct sign inconsistency of Self-Evaluation suggests that a high level of self-monitoring may, on one hand, enhance learners' sense of control over their learning, but on the other, amplify their experience of academic pressure. Guo (2018) noted that in classrooms with strong evaluative orientations, highly self-disciplined individuals are more prone to increased anxiety, as they tend to link self-assessment closely with external expectations. Similarly, the systematic review by Broadbent and Poon (2015) found that some self-regulated learners experience greater emotional burden due to excessive self-reflection and perfectionism. In line with Pekrun's (2006) Control–Value Theory, it is posited that when learners hold both high task value and a strong sense of control, overly demanding performance evaluations can trigger negative emotions such as anxiety. The current findings are consistent with this dual-edged mechanism of “high monitoring–high pressure.” However, this pattern should be interpreted with caution, as it may partly reflect a model-dependent suppressor-like statistical pattern rather than a direct psychological mechanism or a formally verified suppression effect. Specifically, intrinsic motivation was negatively correlated with anxiety at the bivariate level but positively predicted anxiety in the multivariate model, suggesting that the observed indirect pathway may be contingent on shared variance among highly correlated predictors rather than representing a simple causal process.

The full mediation effect of Goal Setting on anxiety via intrinsic motivation suggests that the quality of motivation serves as a key intermediary in emotion-related outcomes. Zimmerman (2002) identified goal setting as the starting point of self-regulated learning, which influences learning engagement and emotional regulation by stimulating motivation. Elliot and McGregor's (2001) Achievement Goal Theory further suggests that both performance-approach and performance-avoidance goals can induce anxiety in high-expectation contexts. The present findings align with those of Xu et al. (2022), who found that high-quality goal setting reduced anxiety by enhancing autonomous motivation among online Chinese learners. Therefore, the emotional impact of goal setting appears to operate not through direct behavioral control but through a motivational reconfiguration process. Nevertheless, given the cross-sectional design of this study, this pathway should be interpreted as an observed association rather than a confirmed causal mechanism.

The direct negative effect of Help Seeking on English Class Performance Anxiety highlights the importance of social support as a contextual buffer in online learning. Research by Lin et al. (2021) shows that learners' anxiety significantly decreases when receiving timely feedback from peers and instructors in online settings. Demirtaş et al. (2015) also emphasized the buffering role of social support in managing stress and emotional responses. Moreover, Raver et al. (2007), from the perspective of emotional regulation in childhood, suggested that social support enhances emotional understanding and regulation, promoting academic adjustment. In line with Law's (2021) study of Chinese international students in cross-cultural contexts, establishing accessible channels for help-seeking and emotional expression can alleviate anxiety and improve mental health. These findings suggest that help-seeking behaviors are not merely learning strategies but also function as emotional regulation resources within digital learning environments.

One particularly noteworthy finding is that intrinsic motivation positively predicted English learning anxiety in the final model, which appears counterintuitive from a conventional SDT perspective. This result should not be interpreted as evidence that intrinsic motivation itself increases anxiety or that intrinsic motivation is maladaptive *per se*. Nor should it be understood as contradicting Self-Determination Theory (SDT). Rather, it more likely reflects a model-dependent statistical association observed under the simultaneous inclusion of correlated predictors within a highly evaluative learning context. First, from a statistical perspective, the coexistence of a negative bivariate correlation and a positive regression coefficient suggests an indirect/direct sign inconsistency that may reflect a suppressor-like pattern, indicating that intrinsic motivation may capture variance related to performance pressure when controlling for overlapping self-regulated learning dimensions. Second, from a contextual perspective, in highly evaluative English learning environments—such as those characterized by test-oriented assessment, social comparison, and performance expectations—students who are more intrinsically engaged may also become more sensitive to performance demands and potential failure. In such contexts, enjoyment of learning and concern about achievement may coexist, leading to a complex motivation–emotion pattern. This interpretation may be particularly relevant in the Chinese educational context and should be understood as a plausible contextual explanation rather than an empirically verified conclusion, since no cultural variables were directly measured in the present study. Therefore, the observed positive association should be understood as a context-specific and model-dependent pattern rather than a generalizable contradiction to SDT.

Overall, this study contributes to the literature in several important ways. First, it demonstrates that self-regulated learning does not exert uniform emotional effects, but instead operates through heterogeneous pathways across different dimensions. Second, it extends SDT by highlighting that the emotional consequences of intrinsic motivation may vary depending on contextual pressures and statistical conditions. Third, it integrates Social Cognitive Theory as the foundation for understanding self-regulation processes, while using Achievement Goal Theory and Control–Value Theory as complementary frameworks to explain the emergence of anxiety under evaluative conditions. Together, these findings provide a more nuanced understanding of the interplay between self-regulation, motivation, and emotion in digital learning contexts.

Although the mediation models reached statistical significance, the practical magnitude of the findings should be interpreted cautiously. In particular, the final model explained 7.9% of the variance in English learning anxiety, indicating that self-regulated learning dimensions and intrinsic motivation represent only part of the broader set of factors influencing anxiety. Other contextual variables—such as teacher support, peer comparison, academic pressure, and prior language proficiency—may play equally or even more substantial roles. Therefore, the present findings should be understood as theoretically meaningful but modest in practical effect size.

This study possesses several strengths. First, it was conducted within the realistic context of China's ongoing digital higher education reform, with a large and geographically diverse sample, enhancing ecological validity. Second, the study adopted a multidimensional approach to self-regulated learning and employed bootstrapped mediation analysis, ensuring methodological robustness. Third, by focusing on emotional outcomes rather than solely academic performance, this study broadens the scope of research on self-regulated learning.

Nonetheless, several limitations should be acknowledged. First, the cross-sectional design limits causal inference, and future research should adopt longitudinal or experimental designs to further examine the temporal dynamics among self-regulation, motivation, and anxiety. Second, reliance on self-reported measures may introduce potential biases, and future studies could incorporate behavioral or physiological indicators to enhance measurement validity. Third, although this study focused on intrinsic motivation, it did not distinguish among its subcomponents; future research could adopt a more fine-grained SDT framework. Fourth, the sample exhibited an uneven demographic distribution, with a predominance of female students and freshmen, which may limit generalizability. Finally, the sample consisted primarily of Chinese university students, and cultural factors such as social comparison and face concerns may have influenced the observed patterns. However, because these cultural variables were not directly measured, such interpretations should remain tentative and should not be treated as empirical conclusions. Future studies could examine these relationships across different cultural contexts to enhance external validity.

## 5 Conclusion

In conclusion, this study revealed the complex interplay between online self-regulated English learning, intrinsic motivation for English learning, and English Class Performance Anxiety. Specifically, Self-Evaluation exhibited a model-dependent indirect/direct sign inconsistency, reflecting a coexistence of motivational activation and emotional burden; Goal Setting was fully mediated through intrinsic motivation, underscoring the importance of goal–motivation alignment; and Help Seeking directly alleviated anxiety via social support mechanisms.

These findings suggest that the effects of self-regulated learning on anxiety are not uniform, but depend on the specific dimensions of self-regulation and their associated motivational pathways. The findings underscore that, in digital foreign language learning environments, the key lies not in the intensity of motivation but in its quality and emotional safety.

From a theoretical perspective, this study contributes to the literature by demonstrating that intrinsic motivation may play differentiated and context-dependent roles in the relationship between self-regulated learning and emotional outcomes. From a practical perspective, the results highlight the importance of designing learning environments that balance motivational enhancement with emotional support. At the same time, given the modest explained variance observed in the final model, these practical implications should be interpreted cautiously, as anxiety is influenced by multiple contextual and individual factors beyond self-regulated learning and motivation alone.

Future pedagogical design should promote dimension-specific, autonomy-supportive SRL interventions that foster positive motivation and robust social support systems to enhance learners' academic wellbeing and mental health. At the same time, these findings should be interpreted as associative rather than causal, and future research is needed to further examine these relationships using longitudinal or experimental designs.

## Data Availability

The raw data supporting the conclusions of this article will be made available by the authors, without undue reservation.
